# Fibroblast growth factor receptor 4 induced resistance to radiation therapy in colorectal cancer

**DOI:** 10.18632/oncotarget.12099

**Published:** 2016-09-17

**Authors:** Mohamed A. Ahmed, Edgar Selzer, Wolfgang Dörr, Gerd Jomrich, Felix Harpain, Gerd R. Silberhumer, Leonhard Müllauer, Klaus Holzmann, Bettina Grasl-Kraupp, Michael Grusch, Walter Berger, Brigitte Marian

**Affiliations:** ^1^ Institute of Cancer Research, Department of Medicine I, Medical University of Vienna, Austria; ^2^ Radiation Biology Department, National Center for Radiation Research and Technology, Egyptian Atomic Energy Authority, Egypt; ^3^ Department of Radiotherapy and Radiobiology, Medical University of Vienna, Austria; ^4^ Christian Doppler Laboratory for Medical Radiation Research for Radiation Oncology, Medical University of Vienna, Austria; ^5^ Department of Surgery, Medical University Vienna, Austria; ^6^ Clinical Institute of Pathology, Medical University Vienna, Austria

**Keywords:** FGFR4, colorectal cancer, radiotherapy, RAD51

## Abstract

In colorectal cancer (CRC), fibroblast growth factor receptor 4 (FGFR4) is upregulated and acts as an oncogene. This study investigated the impact of this receptor on the response to neoadjuvant radiotherapy by analyzing its levels in rectal tumors of patients with different responses to the therapy. Cellular mechanisms of FGFR4-induced radioresistance were analyzed by silencing or over-expressing FGFR4 in CRC cell line models. Our findings showed that the FGFR4 staining score was significantly higher in pre-treatment biopsies of non-responsive than responsive patients. Similarly, high expression of FGFR4 inhibited radiation response in cell line models. Silencing or inhibition of FGFR4 resulted in a reduction of RAD51 levels and decreased survival in radioresistant HT29 cells. Increased RAD51 expression rescued cells in the siFGFR4-group. In radiosensitive SW480 and DLD1 cells, enforced expression of FGFR4 stabilized RAD51 protein levels resulting in enhanced clearance of γ-H2AX foci and increased cell survival in the mismatch repair (MMR)-proficient SW480 cells. MMR-deficient DLD1 cells are defective in homologous recombination repair and no FGFR4-induced radioresistance was observed. Based on our results, FGFR4 may serve as a predictive marker to select CRC patients with MMR-proficient tumors who may benefit from pre-operative radiotherapy.

## INTRODUCTION

Despite technical and therapeutic improvements in recent years, colorectal cancer (CRC) remains one of the most deadly cancers worldwide, in both men and women. Radiotherapy is an integral part of the management strategies for colorectal cancer, especially as a neoadjuvant treatment for locally advanced stage II and III rectal cancer. However, the efficiency of radiotherapy in the treatment of rectal cancer varies significantly between different patients [1]. The mechanistic basis for this intrinsic resistance may be found in differences in DNA-repair and/ or survival processes [2].

In response to radiation-induced double strand breaks (DSBs), the histone variant, H2AX, is rapidly phosphorylated as the first step in recruiting DNA repair proteins [3] – most importantly RAD51, the central catalyst of the error-free homologous recombination (HR) repair [4]. RAD51-dependent HR repair significantly contributes to cell survival and induces cellular resistance to ionizing radiation [5, 6].

The fibroblast growth factor receptor (FGFR) family is a class of receptor tyrosine kinases (RTKs) that includes four highly conserved receptors (FGFR1-4) [7]. FGFRs are known to play crucial roles in tumor cell proliferation, angiogenesis, migration and survival [8], and are overexpressed or over-activated in many human cancers [9–13]. Increased FGFR expression and/or activity has also been reported to play a role in treatment resistance towards both conventional and EGFR-targeting strategies [14–16]. With regard to radiation therapy, inhibition of FGFR1 was found to increase radiation-induced cell killing of mesothelioma cells [17], and targeting FGFR3 enhanced radiation-response in squamous cell carcinomas [18]. In rectal cancer patients, Li et al. [19] showed a correlation between high FGFR2 expression and poor therapeutic response to neoadjuvant chemoradiation. By contrast, restoration of FGFR2 enhanced radiosensitivty of prostate cancer cells by increasing apoptosis [20].

FGFR4 was found to be up-regulated in about 25% of all CRC cases and showed oncogenic potential in cell line models of CRC [13]. FGFR4 expression was found to be upregulated in apoptosis-resistant clones after exposure to DNA-damaging agents [21]. Furthermore, FGFR4 silencing resulted in decreased activity of pro-survival signaling, expression of the anti-apoptotic proteins, and showed synergistic interaction with 5-fluorouracil (5-FU) and oxaliplatin in colon cancer cell lines [22].

Here, we investigated for the first time the role of FGFR4 in the resistance of colorectal cancer cells to radiotherapy, and the possible mechanisms of interaction with the DNA damage response machinery (DDR). Our findings indicate that targeting FGFR4 induces radiosensitization that is associated with the attenuation of DSB repair by RAD51-mediated homologous recombination.

## RESULTS

### FGFR4 correlates with poor clinical outcome in neoadjuvant chemoradiation-treated rectal cancer patients

For 43 patients who received neoadjuvant therapy, pre-treatment biopsies were available for analysis. The patients were 28% female and 72% male and their median age was 68 years (Table 1). The majority suffered from locally advanced tumors (40/43 patients; 93%) with affected lymph nodes (30/43 patients; 69.7%). The neoadjuvant treatment caused a reduction of tumor size in 18 patients (41.8%) and a decrease of node involvement in 17 patients (39.5%). Complete remission (stage 0) was observed in 4 (9.3%) cases. Pathological response was determined based on the presence of viable tumor cells in the tissue specimen after surgery [23].

**Table 1:** Patient cohort

Age, median (range)	68 (26–90)
**Sex, *n* (%)**	
Female	12 (28)
Male	31 (72)
**Pre-treatment staging *n* (%)**	
T1, 2	3 (7.0)
T3, 4	40 (93.0)
N0	13 (30.2)
N1, 2	30 (69.8)
Stage I, II	13 (30.2)
Stage III	30 (69.8)
**Post-treatment staging, *n* (%)**	
ypT1, 2	21 (48.8)
ypT3, 4	22 (51.2)
ypN0	30 (69.8)
ypN1, 2	13 (30.2)
Stage 0	4 (9.3)
Stage I, II	26 (60.5)
Stage III	13 (30.2)

**Figure 1:**
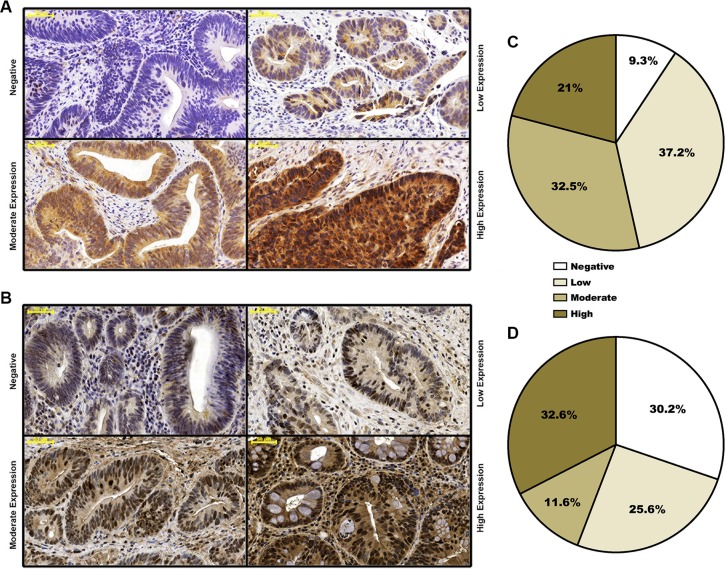
Distribution of staining-based FGFR4 and RAD51 expression in pre-neoadjuvant rectal cancer biopsies Representative staining of biopsies exhibiting negative, weak, moderate and strong FGFR4 (**A**) or RAD51 (**B**) staining. Rectal cancer tissues were classified according to overall staining intensity for FGFR4 (**C**) and RAD51 (**D**), based on slide scans and morphometric analysis. Scale bar = 50 μm.

Sections obtained from both the pre-treatment biopsies and the surgical specimens were stained to determine FGFR4 and RAD51 protein levels. Representative examples of negative, weak, moderate or strong staining are shown in Figure 1A and 1B. Positive staining was observed in 39/43 (90.7%) cases for FGFR4 and 29/43 (69.8%) cases for RAD51 (Figure 1C and 1D).

**Figure 2:**
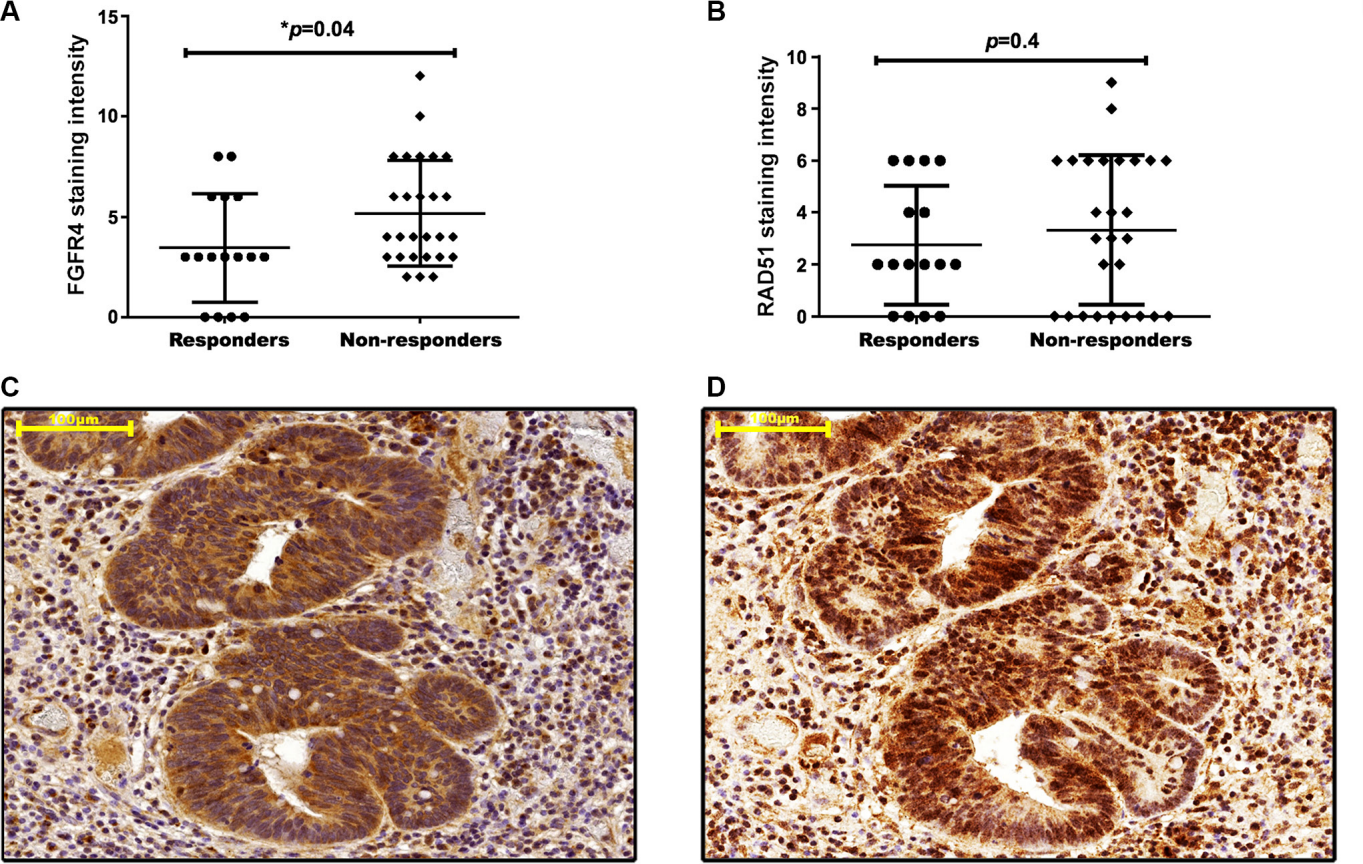
FGFR4 correlates with RAD51 protein levels and poor clinical outcome in human rectal cancer FGFR4 (**A**) and RAD51 (**B**) staining intensity in pre-treatment biopsies was scored for responders and non-responders, according to the immunoreactive scoring (IRS) described in the “materials and methods.” The figures show the individual values together with the mean intensity score ± SEM, *p < 0.05 − *t*-test. Representative staining of FGFR4 (**C**) and RAD51 (**D**) in a resected rectal tumor of a patient who did not respond to the neoadjuvant chemoradiotherapy regimen. Scale bar = 100 μm.

When patients were grouped according to FGFR4 staining intensity, no significant association was observed between FGFR4 expression and gender or age. Analysis with regard to the pre-treatment or post-treatment tumor stage revealed a tentative association with FGFR4 levels that did not achieve statistical significance (Table 2). Downstaging was achieved in 9 patients of the low-FGFR4 group and 7 patients of the high FGFR4 group (*p* > 0.05). 3 of the 4 patients who showed complete clinical response (post-treatment stage 0) were in the low-FGFR4 group. When local response was assessed by the number of viable tumor cells in the surgical specimens, a significant correlation was found: moderate to high expression of FGFR4 was observed in 78.3% of the weakly or non-responsive cases, but in only 21.7% of responsive patients (Table 2; *p* = 0.03 by χ^2^-test). Also FGFR4 expression was significantly lower in patients showing complete or strong response as compared to weakly or non-responsive patients (Figure 2A; *p* = 0.04). No statistically significant difference was observed for RAD51 staining (Figure 2B).

**Table 2:** FGFR4 expression and its correlation to clinicopathological characteristics and response of neoadjuvant chemoradiation treated rectal cancer patients. (a) *t*-test, (b) Chi square test

	FGFR4 Expression		*p*-Value
	(Negative - Weak)*	(Moderate - Strong)*	
**Median age, years**	68.5 (26–79)	67.5 (34–90)	0.22(a)
**Sex, *n* (%)**			
Women	5 (25)	7 (30.43)	0.74(b)
Men	15 (75)	16 (69.56)	
**Pre-treatment grading and staging**			
**Depth of invasion, *n* (%)**			
T1, 2	2 (10)	1 (4.35)	0.59(b)
T3, 4	18 (90)	22 (95.65)	
**Lymph node metastasis, *n* (%)**			
N0	8 (40)	5 (21.74)	0.31(b)
N1, 2	12 (60)	18 (78.26)	
**TNM stage, *n* (%)**			
Stage I, II	8 (40)	5 (21.74)	0.31(b)
Stage III	12 (60)	18 (78.26)	
**Post-treatment grading and staging**			
**Depth of invasion, *n* (%)**			
ypTX, 1, 2	11 (55)	10 (43.48)	0.54(b)
ypT3, 4	9 (45)	13 (56.52)	
**Lymph node metastasis, *n* (%)**			
ypN0	16 (80)	14 (60.86)	0.2(b)
ypN1, 2	4 (20)	9 (39.13)	
**TNM stage, *n* (%)**			
Stage 0	3 (15)	1 (4.35)	
Stage I, II	13 (65)	13 (56.52)	0.25(b)
Stage III	4 (20)	9 (39.13)	
Therapy response**			
Strong response (2–4)	11 (55)	5 (21.74)	0.03(b)
Weak or no response (0–1)	9 (45)	18 (78.26)	

* The classification was done according to the immunoreactive score (IRS): Negative-Weak (0–3), Moderate-High (4–12).

** Response was determined according to the criteria of Dworak et al. [23].

In addition, FGFR4 and RAD51 were analyzed in surgical specimens of non-responsive patients whose tumors were surgically resected after the neoadjuvant treatment. In these tumors a strong co-expression was observed for FGFR4 and RAD51 (Figure 2C and 2D).

### FGFR4 is upregulated in radioresistant HT29 cells in correlation with homologous recombination-regulating proteins

To establish an *in vitro* model for the analysis of the underlying cellular mechanisms we evaluated radiosensitivity of CRC cells using clonogenic survival assays (Supplementary Figure 1A). HT29 cells were significantly less radiosensitive as compared to both SW480 and DLD1 cells, represented by higher radiation ED_50_; 4.42 ± 0.13 Gy for HT29 as compared to 2.6 ± 0.07 Gy for SW480 (*p* < 0.0001) and 2.52 ± 0.12 Gy for DLD1 (*p* < 0.0001). We investigated FGFR4 expression in these cell lines and found that the radioresistant HT29 cells showed 42% (*p* < 0.01) and 85.6% (*p* < 0.0001) higher expression than SW480 and DLD1 cells, respectively, as measured by qPCR (Supplementary Figure 1B). The efficiency of homologous recombination repair in the 3 cell lines was determined by HR reporter assay using GFP-based reporter construct [24], and the efficiency was highest in HT29 cells and lowest in DLD1, significantly and positively correlating with FGFR4 expression (*r* = 0.9, *p* < 0.05; Supplementary Figure 2).

**Figure 3:**
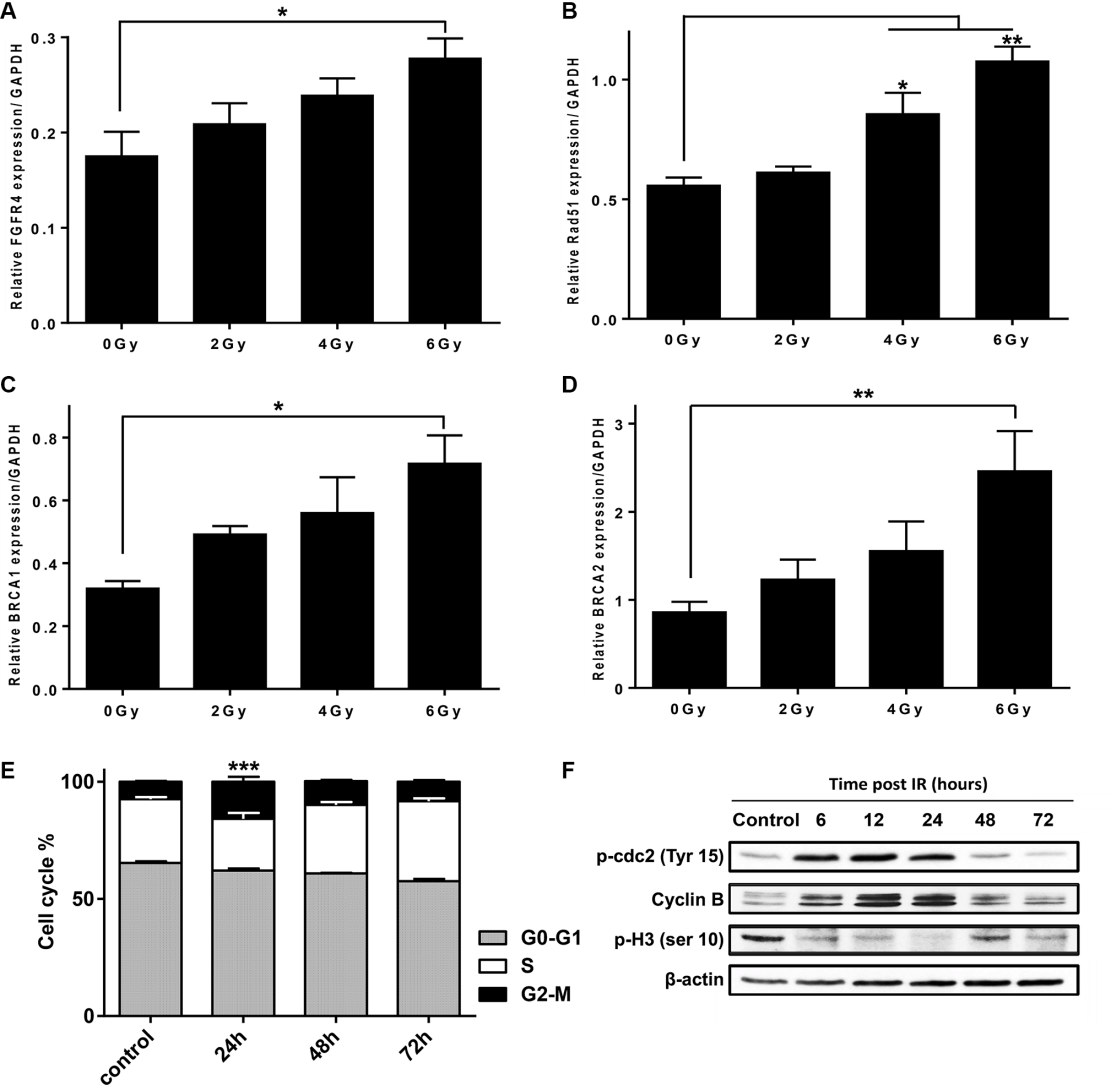
FGFR4 expression is upregulated after irradiation in a dose-dependent manner together with key homologous recombination-related proteins Expression of (**A**) FGFR4, (**B**) RAD51, (**C**) BRCA1 and (**D**) BRCA2 genes were determined by qPCR, 24 h after exposure to different doses of γ-radiation (0, 2, 4 and 6 Gy) in HT29 cells. The expression levels were calculated relative to GAPDH. (**E**) Cell cycle distribution of HT29 cells irradiated with a single 6 Gy dose of γ-rays. Analysis was done using FACS at 24, 48 and 72 hours post irradiation. (**F**) Western blot of cdc2 phosphorylation (Tyr-15) status, Cyclin B1 expression, and the histone H3 phosphorylation (Ser-10) in HT29 cells at different time points after exposure to 6 Gy dose. Beta actin was used as loading control.

24 h after exposure of HT29 cells to γ-rays, FGFR4 mRNA was increased in a dose-dependent manner (Figure 3A) and was 1.6-fold higher than the mock-irradiated control after a 6 Gy dose (*p* < 0.05). We also assessed the expression levels of the HR-related proteins: RAD51, BRCA1 and BRCA2, in response to radiation in HT29 cells (Figure 3B–3D). Similar to FGFR4, mRNA-levels of these genes were dose-dependently upregulated by radiation reaching an increase of 1.92-fold (*p* < 0.01), 2.24-fold (*p* < 0.05) and 2.86-fold (*p* < 0.01) compared to non-irradiated cells for RAD51, BRCA1 and BRCA2, respectively.

The cell cycle profile of irradiated HT29 cultures showed 2.1-fold (*p* < 0.001) increases of G2/M fraction 24 h after a single 6 Gy dose, as compared to mock-irradiated cells (Figure 3E). The G2/M arrest was further confirmed by detection of cdc2 carrying a deactivating phosphorylation at Tyr15 (Figure 3F) at 6, 12 and 24 h after IR. In addition, cyclin B levels were increased, while the phosphorylation of histone H3 at Ser-10, a crucial event for the onset of mitosis, was found to drop early after irradiation until complete inhibition at 24 h post irradiation.

### HT29 cells are radiosensitized by RAD51 depletion

**Figure 4:**
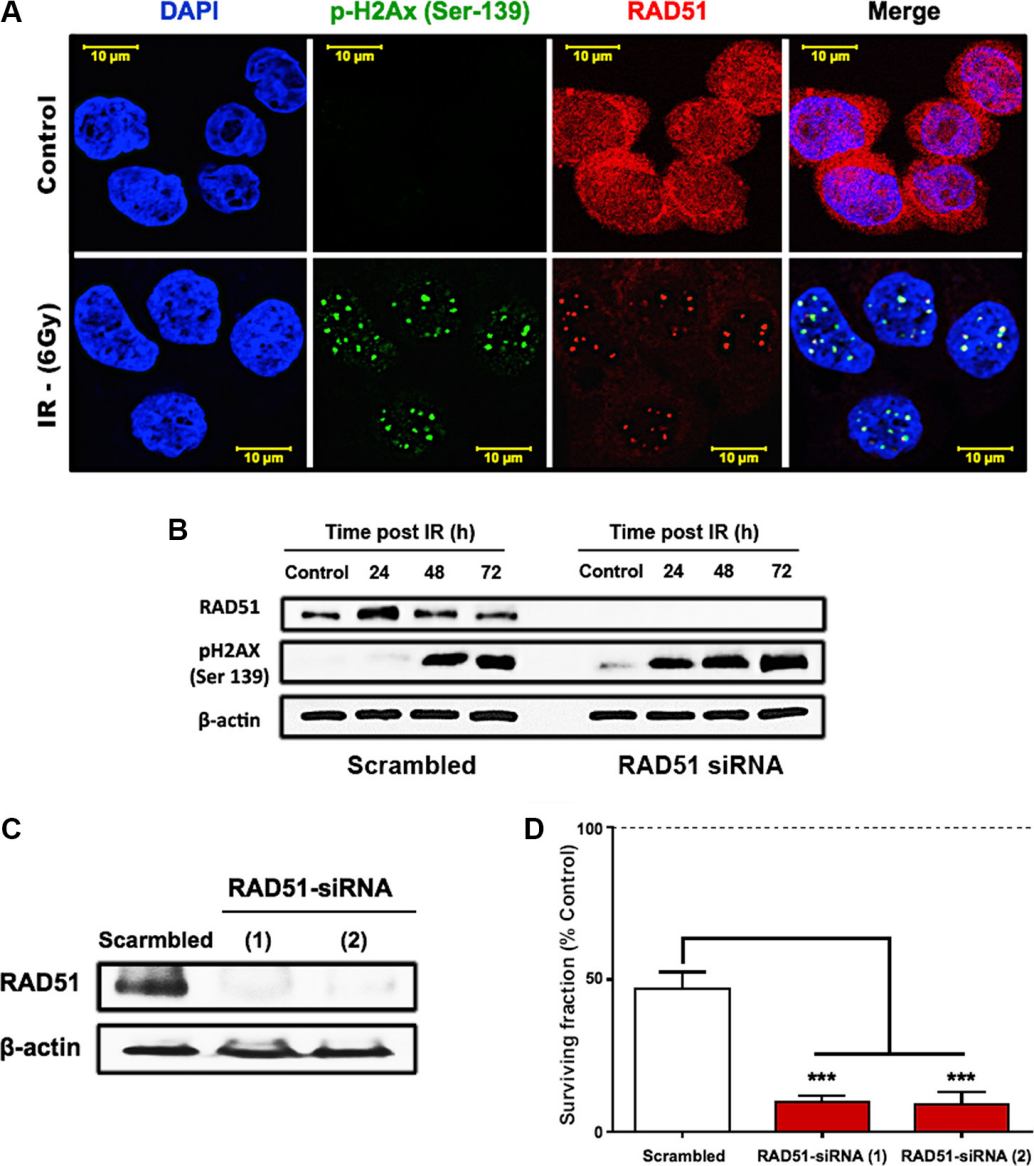
RAD51-dependent HR is a crucial mediator of HT29 survival after irradiation (**A**) Immunofluorescence of RAD51 and γ-H2AX foci formation post IR. HT29 cells were seeded onto cover slips and treated with a single 6 Gy dose of γ-rays. 24 h after IR, cells were formalin fixed, permeabilized, and stained with RAD51 and γ-H2AX antibodies. (**B**) Western blots showing the effect of RAD51 knockdown on the damage marker, γ-H2AX. (**C**) Western blots confirming the efficiency of the two tested RAD51 siRNAs. (**D**) Clonogenic surviving fractions of scrambled/RAD51 siRNA treated HT29 cells showing increased cell killing and induced radiosensitivity of the radioresistant HT29 cells after RAD51 knockdown. Cells were exposed to a single 6 Gy dose of γ-rays, and the surviving fractions were calculated by dividing the number of colonies counted by the corresponding number of cells seeded as described in “Materials and Methods.”

To assess the role of RAD51 in radioresistance of HT29 cells, we performed immunofluorescence staining to observe the localization of RAD51 before and after irradiation with 6 Gy (Figure 4A). In the control cells, RAD51 appeared to be abundant and was localized not only nuclear but also perinuclear. 24 h after exposure to 6 Gy of γ-rays, damage foci were visible when stained for γ-H2AX and RAD51 that was recruited to these repair foci. Also, we investigated the regulation of RAD51 on the protein level by western blotting (Figure 4B) and observed a transient increase of the RAD51 after 24 h followed by a steady return to control levels at 48 and 72 hours. At these later time points unresolved damage became apparent through an increase of the γ-H2AX in the cells (Figure 4B). Knockdown of RAD51 was achieved using siRNA oligonucleotides that efficiently depleted RAD51 expression (Figure 4C). This resulted in higher persistence of γ-H2AX (Figure 4B) and in a significant decrease of survival (Figure 4D, *p* < 0.0001).

### FGFR4 silencing radiosensitized HT29 cells via attenuation of DSB repair by HR

**Figure 5:**
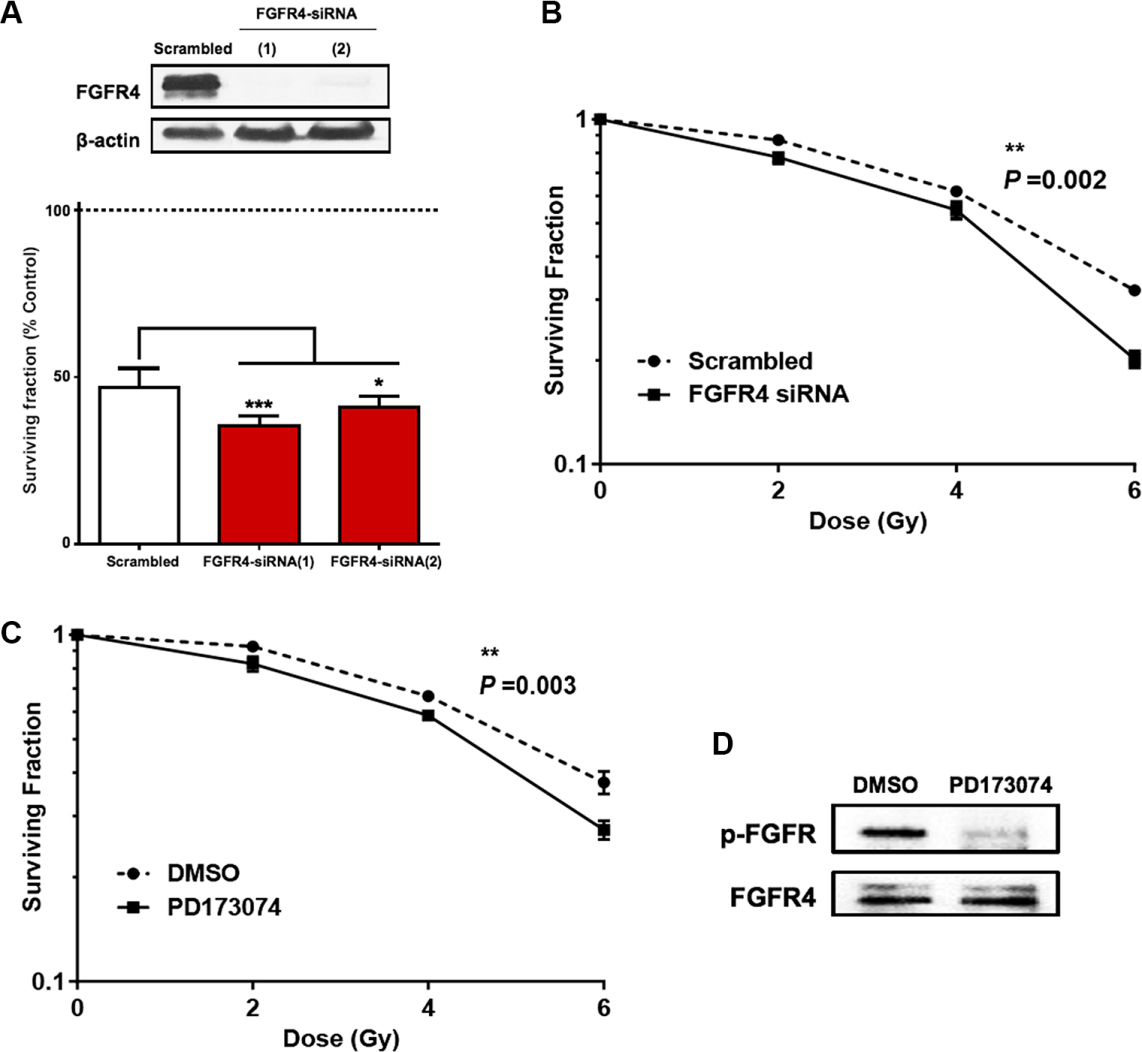
Silencing of FGFR4 induced loss of survival in radioresistant HT29 cells The knockdown of FGFR4 expression in HT29 cells was achieved using siRNA targeting FGFR4 one day before exposure to a single 6 Gy dose of γ-rays. Two different FGFR4 siRNAs were used and the knockdown efficiency was confirmed by western blot (**A**, upper panel) and qRT-PCR (A, lower panel). (**B**) Colony formation assay showed a significant decrease in the surviving fraction of FGFR4-knocked down HT29 cells. (**C**) Exposure of the cells to the FGFR-inhibitor PD173074 3 hours before exposure to a single 6 Gy dose caused a similar decrease in colony formation capacity of HT29 cells. HT29 cultures were treated with 2 μM PD173074 to inhibit FGFR4-dependent signaling. The efficacy of the inhibitor in blocking the phosphorylation and the activation of the receptor was confirmed with western blot (**D**).

To investigate the role of FGFR4 in the radioresistance of HT29 cells, two different strategies were followed. First, we used siRNA-induced FGFR4 silencing (Figure 5A), which caused a significant decrease of the surviving colony forming cells (*p* < 0.01) after radiation (Figure 5B). This is represented by a shift in the dose-response curve and a lower radiation ED_50_ (3.83 ± 0.18 Gy) as compared to scrambled controls (4.6 ± 0.09 Gy). Secondly, we used the FGFR inhibitor PD173074 to block FGFR4-dependent signaling. 2 μM PD173074 were applied 3 h before irradiation and the treatment was continued after irradiation and resulted in a significant reduction of the surviving fraction (*p* < 0.01) as well as 15.3% decrease of the radiation ED_50_ (3.81 ± 0–16 Gy vs. 4.51 ± 0.09 Gy for control) (Figure 5C). Phosphorylation of FGFR4 was effectively prevented by the drug (Figure 5D).

**Figure 6:**
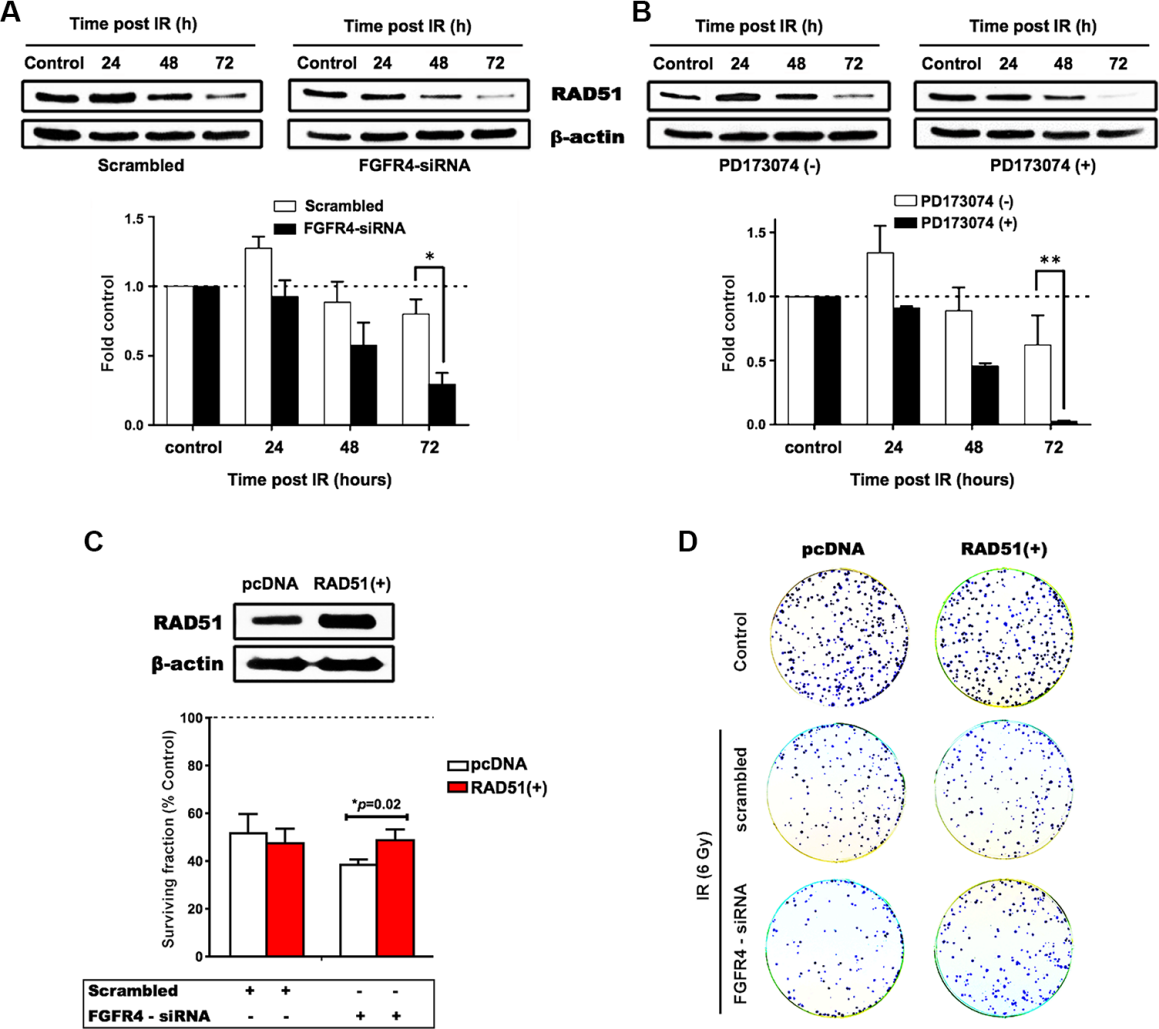
FGFR4-mediated radiation response involves regulation of RAD51 Proteins were isolated at the indicated time points after irradiation from cultures treated with siRNA targeting FGFR4 (**A**) or with the FGFR-inhibitor PD173074 (**B**). The upper panels show typical western blots of RAD51 protein expression in irradiated HT29 cells. The lower panels depict the quantification of RAD51 protein expression from 3 independent experiments normalized to control. (**C**) RAD51 was overexpressed using a RAD51 expressing vector in HT29 cells as confirmed by western blotting (upper panel). HT29 cells seeded into 6 well plates were transiently transfected with a vector expressing RAD51 or the control vector. For determination of radiation response, cells were co-transfected with RAD51 or control vectors together with either scrambled or FGFR4 siRNAs, before exposure to a single 6 Gy dose of γ-rays. The surviving fraction of the transfected cells was measured by quantification of colonies (lower panel). (**D**) Representative images of the clonogenicity assay.

**Figure 7:**
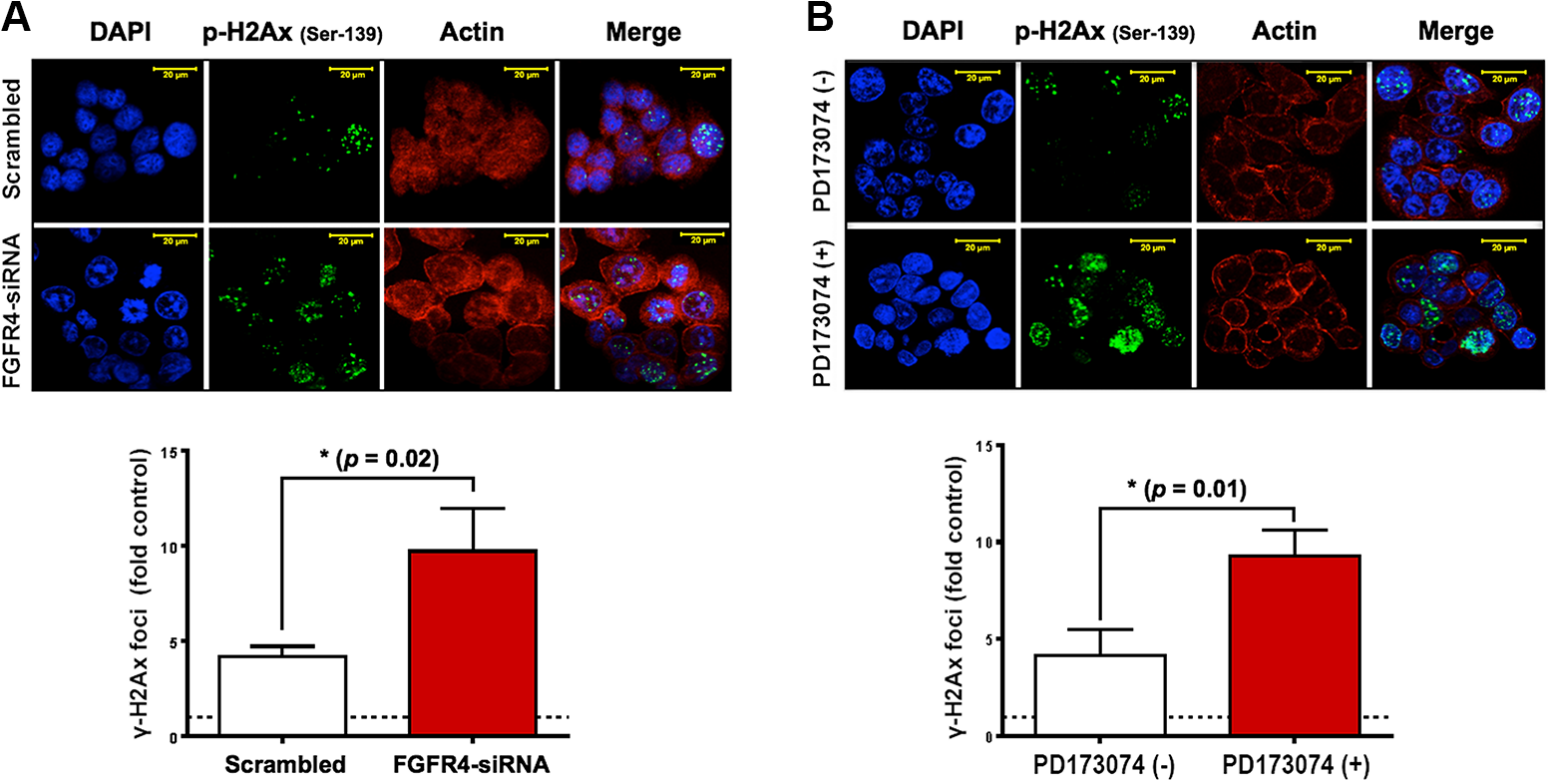
FGFR4 silencing-induced damage persistence in HT29 cells Immunofluorescence of γ-H2AX foci post IR was performed as described in Materials and Methods, and Figure 4. After FGFR4 knockdown (**A**) or PD173074 treatment (**B**), cells were treated with a single 6 Gy dose of γ-rays and stained for γ-H2AX 24 h later. Upper panels show representative photographs, lower panels show the quantification of γ-H2AX-foci.

With regard to RAD51, both FGFR4 depletion and signaling blockade resulted in an accelerated decrease of RAD51 protein levels as determined by western blotting (Figure 6A and 6B). This indicates that the effect of FGFR4 on radiation response is mediated through the regulation of this repair protein. Overexpression of RAD51 controlled by a CMV promoter increased RAD51 levels in HT29 cells (Figure 6C) and also abolished the decrease of cell survival induced by FGFR4 knockdown (*p* < 0.05; Figure 6D). Furthermore, irradiation of FGFR4-silenced HT29 cells resulted in significantly higher γ-H2AX-foci accumulation (*p* < 0.05; Figure 7A and 7B).

### Increased FGFR4 expression increased survival of SW480 cells but not the mismatch repair-deficient DLD1 cells after irradiation

**Figure 8:**
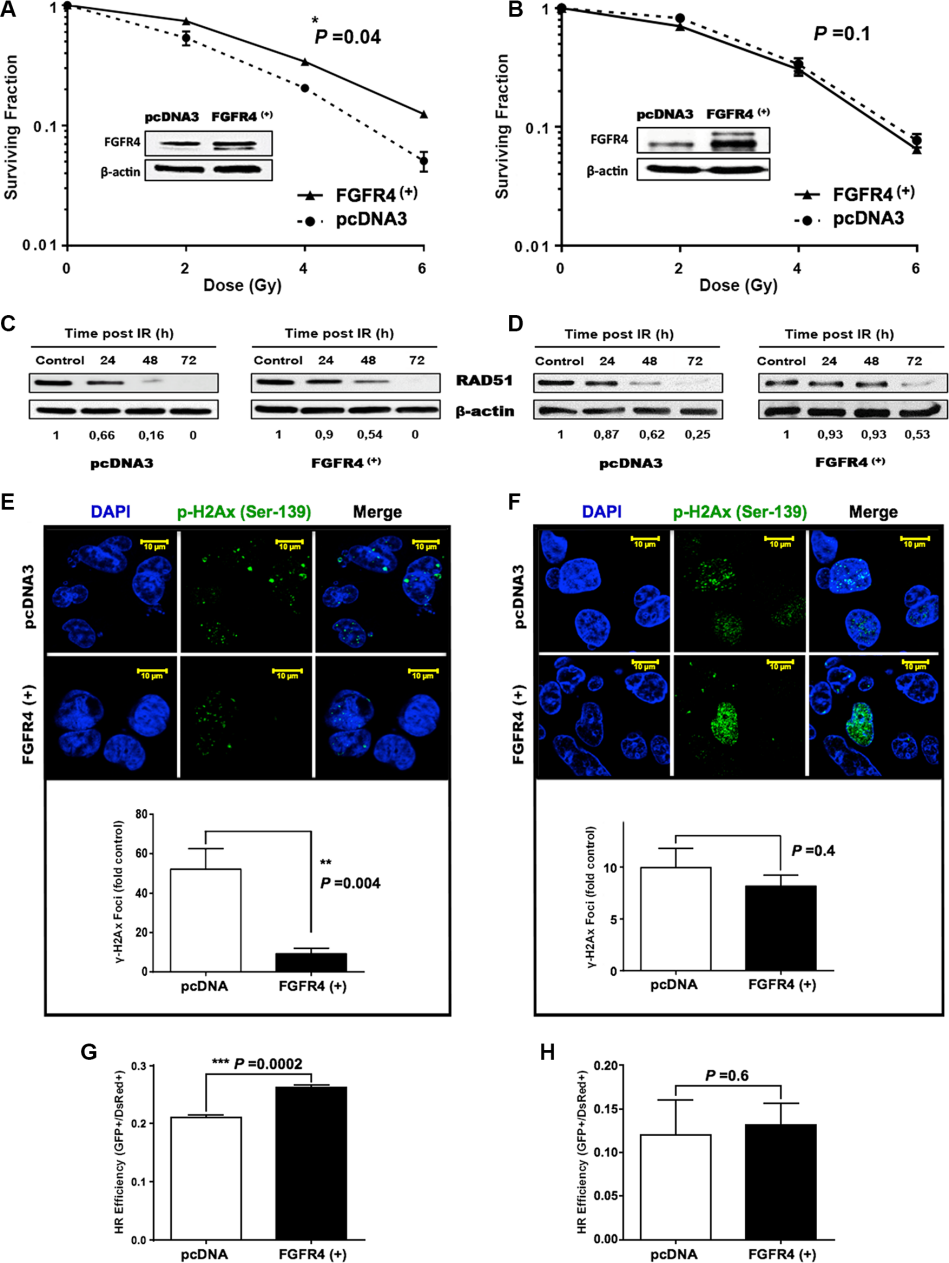
FGFR4 overexpression induced radioresistance FGFR4 was overexpressed in MMR-proficient SW480 cells and in MMR-deficient DLD1 cells and overexpression was verified by western blotting. After γ-irradiation the surviving cell fraction was increased in the FGFR4-SW480 cells (**A**), but not in the FGFR4-DLD1 cells (**B**). Western blot showing stabilization of RAD51 protein levels of irradiated FGFR4 overexpressing SW480 (**C**) and DLD1 (**D**) cells, as compared to pcDNA-transfected cells. RAD51 protein lysates were collected at 24, 48 and 72 h after single 6 Gy dose. Fluorescence staining of γ-H2AX in formalin fixed SW480 (**E**) and DLD1 (**F**) cells 24 h after irradiation with a single 6 Gy dose. Upper panels show representative pictures of γ-H2AX foci (green fluorescence), lower panels show the quantification of γ-H2AX foci relative to control. Quantification of the HR capacity in SW480 (**G**) and DLD1 (**H**) cells, represented by the ratio of GFP+ cells to DsRed+ cells, as described in the Matrials and Methods. The bars represent the mean of 3 independent cultures ± SEM.

To answer the question whether FGFR4 overexpression conveys radioresistance to sensitive cells, FGFR4-overexpressing SW480 and DLD1 cells were obtained. DLD1 were utilized as a model of mismatch repair (MMR) defective cells (microsatellite instable, MSI), while SW480 is a microsatellite-stable (MSS) cell line. Increased FGFR4 expression significantly improved survival of SW480 cells (Figure 8A, *p* < 0.05) and resulted in a 41% increase of radiation ED_50_ (3.01 ± 0.06 Gy vs. 2.13 ± 0.27 Gy for pcDNA3). On the other hand, FGFR4 overexpressing DLD1 cells did not respond with increased cell surviving fraction (Figure 8B).

RAD51 protein levels were stabilized by FGFR4 overexpression in both cell lines (Figure 8C and 8D). Functional activity of the DSB repair appeared fundamentally different, however. In SW480 cells, FGFR4 induced clearance of DNA breaks after irradiation resulting in a significant decrease of persisting nuclear γ-H2AX-foci (Figure 8E, *p <* 0.01). In DLD1 cells the persisting radiation-induced γ-H2AX foci were not reduced (Figure 8F). This was further confirmed by the significant enhancement of the HR-repair capacity, which was exclusively observed in SW480 cells (Figure 8G, *p* = 0.0002), but not DLD1 cells (Figure 8H, *p* = 0.6), upon increased FGFR4 expression.

## DISCUSSION

Overexpression of FGFR4 was observed in several cancers and has been reported to be associated with aggressive tumors and poor prognosis in breast cancer [25], squamous cell carcinoma [26], ovarian cancer [11], non-small cell lung cancer [27], gastric cancer [28], as well as colorectal cancer [13, 22]. It has also been reported to be associated with therapy response [21, 22]. Ionizing radiation is known to induce cell killing through induction of DNA-damage, with double strand breaks (DSBs) being the most fatal. To cope with that, cells have evolved several repair mechanisms, the most important being the error-prone non-homologous end joining (NHEJ), and the error-free homologous recombination (HR). Cancer cells were found to become resistant to radiation by increasing the activity of DNA repair proteins involved in the HR repair machinery [6, 29]. Our work now reports that FGFR4 enhanced the response of human colorectal cancer cells to radiation therapy by upregulating RAD51 and consequently increasing HR capacity.

The results demonstrate that high FGFR4 expression in the tumor correlated with poor response to radiotherapy in 43 patients who underwent neoadjuvant treatment for rectal cancer. Specifically, 3 of the 4 patients who achieved complete clinical response showed only low FGFR4 levels and 78.3% of the specimens with high FGFR4-positive staining were obtained from patients that did not favourably respond to radiotherapy (Table 2) suggesting a predictive value of FGFR4-levels in pre-treatment biopsy specimens. Moreover, the FGFR4-score was shown to be significantly higher in partially and non-responsive patients as compared to those who strongly responded to the neoadjuvant chemoradiation regimen (Figure 2A). For RAD51, we observed a trend to higher protein levels in biopsies from non-responders, however not statistically significant. This may be due to the small cohort we analyzed and the difference may become significant in a higher-powered study. A published report by Tennstedt et al. [30] using a cohort of 1213 CRC patients actually did identify RAD51 as a marker for poor prognosis. However, the endpoint studied was overall survival, while we only assessed the immediate response to neoadjuvant treatment. The fact that our cohort consisted of only rectal cancer patients probably is not critical as Tennstedt et al. did not see differences between the complete cohort and a rectum-only subcohort [30].

Interestingly, we also observed strong co-staining of FGFR4 and RAD51 in surgical specimens of patients who have not responded to neoadjuvant radiotherapy (Figure 2C and 2D) indicating that specifically those tumor cells that expressed high FGFR4 and upregulated RAD51 had survived the radiation treatment. Hence, FGFR4 overexpression may predict neoadjuvant radiotherapy response, serving as an indicator to select CRC patients who could potentially benefit from neoadjuvant radiotherapy.

On the cellular level, we have demonstrated that repair of radiation damage was dependent on RAD51-mediated homologous recombination in the radioresistant HT29 cells. After irradiation, HT29 cells underwent a transient G2/M arrest and transcriptionally upregulated the HR-associated genes RAD51, BRCA1 and BRCA2. RAD51 protein was increased as compared to control cells and was recruited to γ-H2AX-positive damage foci in the nuclei of irradiated cells (Figure 4). This process is known to be restricted to the G2 phase of the cell cycle, where G2-arrest allows time to repair the damage [31]. In our study, the IR-induced G2 arrest was shown by FACS-analysis (Figure 3E), and further demonstrated by an increase in the deactivating Tyr15-phosphorylation of cdc2 (CDK1), increased levels of cyclin B, and decreased phosphorylation of histone H3 (Figure 3F). In addition to halting the cell cycle, this may result in diminished CDK-mediated phosphorylation of BRCA2 – a modification that inhibits HR by impairing the interaction of BRCA2 with RAD51 [32]. In spite of the optimal conditions for HR that were observed in the radiation-resistant HT29 cells, DSB is incomplete so that residual damage accumulated 2–3 days after a 6 Gy dose of γ-irradiation (Figure 4B). After siRNA-mediated RAD51 silencing, the accumulation of residual γ-H2AX was increased over time accompanied by a significant reduction in colony formation capacity after irradiation (Figure 4D, *p* < 0.0001). This confirmed that RAD51 is a crucial promoter of survival in the radioresistant CRC cells.

Previous studies have reported the involvement of tyrosine kinase receptors like epidermal growth factor receptor (EGFR), insulin-like growth factor type 1 receptor (IGF-1R), and hepatocyte growth factor receptor (c-Met), in radiation-induced DNA damage repair by homologous recombination through the regulation of RAD51 [33–36]. We now, introduce FGFR4 as a new candidate receptor capable of mediating radioresistance of CRC cells. FGFR4 expression correlated with HR-repair capacity in our CRC cell models (Supplementary Figure 2). However, over-expression of FGFR4 did not affect the baseline expression of RAD51 in either SW480 or DLD1 cells (unpublished observation). Rather, the radiation-induced expression of the protein was enhanced (Figure 8). FGFR4 was also found to be upregulated in a dose-dependent manner after irradiation, in correlation with the HR-regulating proteins RAD51, BRCA1 and BRCA2 (Figure 3). Silencing of FGFR4 by siRNA-mediated knockdown or inhibition of the FGFR4 kinase significantly lowered RAD51 protein levels and radiosensitized HT29 cells (Figure 5) demonstrating FGFR4-mediated regulation of RAD51 in these cells. Increased RAD51 expression successfully rescued FGFR4-silenced HT29 cells (Figure 6) confirming that RAD51 regulation mediated the FGFR4-induced radioresistance.

However, overexpression of FGFR4 only increased cell survival in the MMR-competent cell line SW480, but not in the MMR-deficient cell line DLD1 (Figure 8). This is in agreement with several studies indicating the involvement of the mismatch repair system in radiation-induced DSB repair. In MMR-deficient CRC cell lines, high sensitivity to γ-irradiation as a result of impaired NHEJ as well as defective HR repair, has been reported by others [37, 38]. It has been demonstrated that the recruitment of RAD51 to the damage sites is delayed in MSH2 deficient cells [39], like DLD1. Also, loss of MSH2 may influence the NHEJ pathway at the step of pairing of terminal DNA tails, as reported [40]. Moreover, expression of MLH1 was found to be induced by irradiation and its loss resulted in increased cell cycle progression plus increased radiation-induced chromosomal translocations [41]. Finally, a significant negative correlation has been observed between RAD51 expression and the loss of the MMR proteins, MSH and MLH [30]. Our results demonstrate that similar levels of RAD51 protein caused a significant increase of γ-H2AX-foci clearance capability of FGFR4-overexpressing SW480 cells but not of FGFR4-overexpressing DLD1 (Figure 8C and 8F). As persistence of γ-H2AX foci marks delayed repair and correlates with radiosensitivity [42–44], the lack of γ-H2AX-foci clearance in DLD1 cultures demonstrated the functional inefficiency of the RAD51-dependent HR repair in the MMR-deficient cells. This was further proven by using a fluorescence-based homologous recombination repair construct, which showed significant increase of the repair capacity of FGFR4-SW480 cells, but not FGFR4-DLD1 cells (Figure 8G and 8H). In view of our results as well as the mechanistic data discussed above, upregulation of RAD51 after irradiation in tumors lacking mismatch repair proteins reported by Tennstedt et al. [30] may be the result of a compensatory reaction to the impairment of HR.

In summary, our data suggest that overexpression of FGFR4 induced radioresistance by promoting resolution of radiation-induced strand breaks and tumor cell survival exclusively in the mismatch repair-proficient CRC cells but not the mismatch repair-deficient ones. Thus we define a new role for FGFR4 as regulator of radiation-induced DSB repair in colorectal cancer, making it a candidate predictive marker that identifies those patients who may best profit from neoadjuvant chemoradiation. It may also be a candidate target for innovative combination therapies to increase radiation response.

## MATERIALS AND METHODS

### Cell lines

Human colorectal cancer cell lines, SW480 and HT29, were obtained from the American Type Culture Collection. DLD1 was obtained from European Culture Collections. The cell lines were kept under standard culture conditions using minimal essential medium containing 10% FCS (Sigma-Aldrich, St. Louis, USA) under standard tissue culture conditions (5% CO_2_ at 37°C). All the cell lines were authenticated by Eurofins (Vienna-Austria).

### Ionizing radiation and *in vitro* radiosensitivity assay

Cells were irradiated with different doses of γ-radiation (2, 4 and 6 Gy) using a Co-60 radiotherapy unit (Theratron 760, Theratronics, Ottawa, Canada). The surviving fraction of cells was determined by the clonogenic assay and calculated relative to the non-irradiated mock control [45].

### Homologous recombination repair assay

The analysis of homologous recombinaion-mediated DSB repair was performed using chromosomally integrated fluorescent reporter construct, kindly provided by Dr. Andrei Seluanov, as previously described [24]. The assay based on the restoration of normal GFP gene after repair of I-SceI-induced DSB within the GFP-Pem1 gene, which designed to be exclusively repaired by HR. The measured GFP signal by FACS correlates with the HR repair capability of the cells. DsRed was used as indicator of the transfection efficiency.

### RNA isolation and quantitative real-time PCR assay

Total cellular RNA was isolated using Trifast (PeqLab, Germany) reagent according to the manufacturer's instructions, and the mRNA reversely transcribed into cDNA. Reverse transcription products were amplified using TaqMan-based assay performed using the ABI 7500 fast real-time PCR system (Applied Biosystems, Foster City, California, USA), as previously described [46].

### Knockdown of gene expression

Expression of FGFR4 and RAD51 genes were knocked-down by transfection of small interfering siRNAs (FGFR4: ID S5177 and S5176; RAD51: ID S11736 and 4467, Ambion, Austin, TX, USA) using SilentFect (Bio-Rad). Control cells were treated with scrambled siRNA (ID 4390843, Ambion, USA).

### Establishment of a stable FGFR4-expressing cell line

Stable overexpression of FGFR4 was achieved by transfection of SW480 and DLD1 cells with a plasmid expressing wild-type FGFR4 using TransFectin reagent (Bio-Rad, USA), and selection of over-expressors with geneticin (G418, PAA, Pasching, Austria) as described previously [13]. Control cells received pcDNA3 vector DNA (Invitrogen).

### Overexpression of RAD51

Increased expression of RAD51 in HT29 cells plated in 6 well plates was done using a pCMV6-XL4 vector expressing RAD51 (ID SC309019, Origene, USA) that was introduced into the cells using SilentFect (BioRad).

### Protein isolation and western blotting

Protein was extracted using HEPES lysis buffer supplemented with protease inhibitors cocktail (Complete – Roche, Germany) and phosphatase inhibitors. The protein concentration was determined using the Bradford assay (Bio-Rad, Germany). Proteins were analyzed by western blotting. The antibodies used are listed in Supplementary Table 1. Detection was performed using ECL Western Blot Detection Reagents (GE Healthcare).

### Flow cytometry

For cell cycle analysis, cells were harvested at the indicated time point after irradiation. Nuclei were isolated, stained with propidium iodide and analyzed using a FACS-Calibur (BD, Franklin Lakes, NJ, USA), as described previously [47].

### Immunofluorescence

Cells were seeded onto coverslips and fixed using Histofix 4% (Sigma). Fixed cells were permeabilized using 0.2% Triton X100 in PBS and incubated with p-H2AX (Ser139) rabbit monoclonal antibody (Cell Signaling) and/or RAD51 mouse polyclonal antibody (Abnova) (see Supplementary Table 1). After secondary labeling with Alexa 488 conjugated goat anti-rabbit and/or TRITC-conjugated goat anti-mouse antibodies, slides were washed 3 times in PBS. Coverslips were mounted using DAPI containing Vectashield^®^, sealed in polyurethane and stored at 4°C in the dark. Confocal fluorescent images were obtained using a Zeiss LSM 700 confocal microscope (Carl Zeiss, Germany), with a 63× objective.

### Patients and clinical samples

Biopsy specimens were collected retrospectively from 43 patients with rectal cancer who received neoadjuvant chemoradiation treatment at the General Hospital of Vienna during the years 2012–2014. The patients gave their informed consent, and biopsies were taken during colonoscopic examination before preoperative radiotherapy. Tumor specimens were also collected at surgery. The study protocol was approved by the ethics committee of the Medical University of Vienna. All the patients received neoadjuvant regimen of Xeloda^®^ (capecitabine) plus a total of 50 Gy dose. The response to radiotherapy was determined by histopathological examination of surgically resected specimens and classified according to the amount of viable tumor cells in the resected tissue, as described by Dworak et al. [23]. Specifically, 0 – no regression; 1 – dominant tumor mass with few signs of fibrosis; 2 – dominantly fibrotic material with few tumor cells or groups; 3 – very few tumor cells in fibrotic tissue; 4 – complete response - no tumor cells, only fibrotic mass.

### Immunohistochemistry

FGFR4 staining was carried out according to a standard immunohistochemistry (IHC) protocol [48] using polyclonal rabbit-anti-FGFR4 antibody C-16 (Santa Cruz, CA), or mouse polyclonal anti RAD51 antibody (Abnova) (see Supplementary Table 1). The stained slides were scanned with a Panoramic Midi automated slide scanner (3DHISTECH, Hungary). Quantification of positive cells and staining intensity of the FGFR4 and RAD51 stained biopsies and tumor tissue samples was done using Definiens' TissueMap^®^ software.

Evaluation was performed according to immunoreactive score (IRS) by grading staining intensity from 0 (negative), 1 (weak), 2 (moderate), to 3 (strong) (Figure 1), and percentage of positive cells was scored as 0 (negative), 1 (< 25), 2 (26–50%), 3 (51–75%), and 4 (> 75%). The two scores were multiplied to yield immunoreactive score (IRS) values.

### Statistical analysis

Unless otherwise stated, results are presented as mean values ± SEM for three replicate experiments. Data were analyzed by student's *t*-test using GraphPad Prism software (GraphPad, San Diego, CA, USA). Alternatively, one-way ANOVA/Pearson's chi-square test was used for analyzing the association between FGFR4 expression and clinicopathologic parameters. A *p*-value of < 0.05 was regarded significant (*p < 0.05, **p < 0.01, ***p < 0.001).

## SUPPLEMENTARY MATERIALS


