# Correction: Fibroblast growth factor receptor 4 induced resistance to radiation therapy in colorectal cancer

**DOI:** 10.18632/oncotarget.27186

**Published:** 2019-09-03

**Authors:** Mohamed A. Ahmed, Edgar Selzer, Wolfgang Dörr, Gerd Jomrich, Felix Harpain, Gerd R. Silberhumer, Leonhard Müllauer, Klaus Holzmann, Bettina Grasl-Kraupp, Michael Grusch, Walter Berger, Brigitte Marian

**Affiliations:** ^1^ Institute of Cancer Research, Department of Medicine I, Medical University of Vienna, Austria; ^2^ Radiation Biology Department, National Center for Radiation Research and Technology, Egyptian Atomic Energy Authority, Egypt; ^3^ Department of Radiotherapy and Radiobiology, Medical University of Vienna, Austria; ^4^ Christian Doppler Laboratory for Medical Radiation Research for Radiation Oncology, Medical University of Vienna, Austria; ^5^ Department of Surgery, Medical University Vienna, Austria; ^6^ Clinical Institute of Pathology, Medical University Vienna, Austria

**This article has been corrected:** While analyzing the same patient cohort for different markers, the authors realized a mistake in Table 2. The table reports on the correlation of radiation therapy response and FGFR4 immunoreactivity score. For both data sets, the stratification criteria were more stringent than mentioned in the table. However, the numerical results reported in the table were re-checked and found to be correct. The corrected Table 2 is shown below. The authors declare that these corrections do not change the results or conclusions of this paper.

Original article: Oncotarget. 2016; 7:69976–69990. 69976-69990. https://doi.org/10.18632/oncotarget.12099

**Table 2:** FGFR4 expression and its correlation to clinicopathological characteristics and response of neoadjuvant chemoradiation treated rectal cancer patients. (a) *t*-test, (b) Chi square test.

	FGFR4 Expression		*p*-Value **
	(negative - weak)*	(moderate - strong)*	
**Median age, years**	68.5 (26–79)	67.5 (34–90)	0.22 ^(a)^
**Sex, *n* (%)**			
Women	5 (25)	7 (30.43)	0.74 ^(b)^
Men	15 (75)	16 (69.56)	
**Pre-treatment grading and staging**			
**Depth of invasion, *n* (%)**			
T1, 2	2 (10)	1 (4.35)	0.59 ^(b)^
T3, 4	18 (90)	22 (95.65)	
**Lymph node metastasis, *n* (%)**			
N0	8 (40)	5 (21.74)	0.31 ^(b)^
N1, 2	12 (60)	18 (78.26)	
**TNM stage, *n* (%)**			
Stage I, II	8 (40)	5 (21.74)	0.31 ^(b)^
Stage III	12 (60)	18 (78.26)	
**Post-treatment grading and staging**			
**Depth of invasion, *n* (%)**			
ypTX, 1, 2	11 (55)	10 (43.48)	0.54 ^(b)^
ypT3, 4	9 (45)	13 (56.52)	
**Lymph node metastasis, *n* (%)**			
ypN0	16 (80)	14 (60.86)	0.2 ^(b)^
ypN1, 2	4 (20)	9 (39.13)	
**TNM stage, *n* (%)**			
Stage 0	3 (15)	1 (4.35)	0.25 ^(b)^
Stage I, II	13 (65)	13 (56.52)	
Stage III	4 (20)	9 (39.13)	
Therapy response **			
Strong response (3-4)	11 (55)	5 (21.74)	0.03 ^(b)*^
Weak or no response (0-2)	9 (45)	18 (78.26)	

*The classification was done according to IRS: negative-weak (0-2); moderate-strong (3-12) **Response was determined according to the criteria of Dworak et al. [23].

