# Supplementary material for: Fibroblast growth factor receptor 4 induced resistance to radiation therapy in colorectal cancer
Source: Oncotarget. 2016 Sep 17;7(43):69976–90. doi: 10.18632/oncotarget.12099 (PMC5342528; doi:10.18632/oncotarget.12099)
Supplement: Supplementary file 1 [file oncotarget-07-69976-s001.pdf]

Fibroblast growth factor receptor 4 induced resistance to radiation therapy in colorectal cancer

Supplementary Materials

In vitro model cell lines

The CRC cell lines HT29, SW480 and DLD1 were chosen for investigating the cellular mechanisms mediating the impact of FGFR4 on radiation sensitivity. Among these cell lines, HT29 is the most resistant to irradiation as well as the cell line expressing the highest level of FGFR4. SW480 and DLD1 are more sensitive to radiation and express less of the receptor.

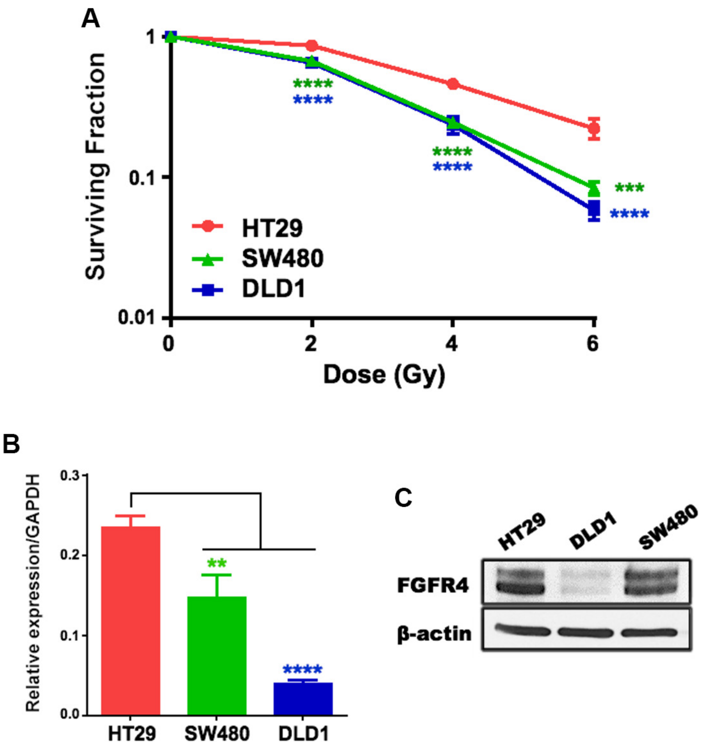

Supplementary Figure S1: (**A**) Clonogenic survival assay showing radiation survival advantage of HT29 cells over DLD1 and SW480 cells. HT29, DLD1 and SW480 cells were subjected to various doses of ionizing radiation (0–6 Gy). Results are presented as mean values ± SEM for at least three replicate experiments (**$p < 0.01$, ***$p < 0.001$, ****$p < 0.0001$), two-way ANOVA analysis. (**B**) Real-time PCR data showing the mRNA expression of FGFR4 in the 3 cell lines. Expression levels were represented by normalizing against GAPDH (analysed by *t*-test: **$p < 0.01$, ****$p < 0.0001$). (**C**) Western blot showing FGFR4 protein expression status in the test cell lines. Equal loading was assessed by probing for beta actin.

Homologous recombination capacity of the cells

To evaluate the cellular capacity to repair the DSBs via homologous recombination repair machinery, cells were transfected with a mixture of I-SecI expression vector and GFP-based reporter cassette that was kindly given by Dr. Andrei Seluanov, as previously described [24]. The relative efficiency of DNA double strand break repair by homologous recombination is represented as the ratio of GFP+ cells to DsRed+ cells.

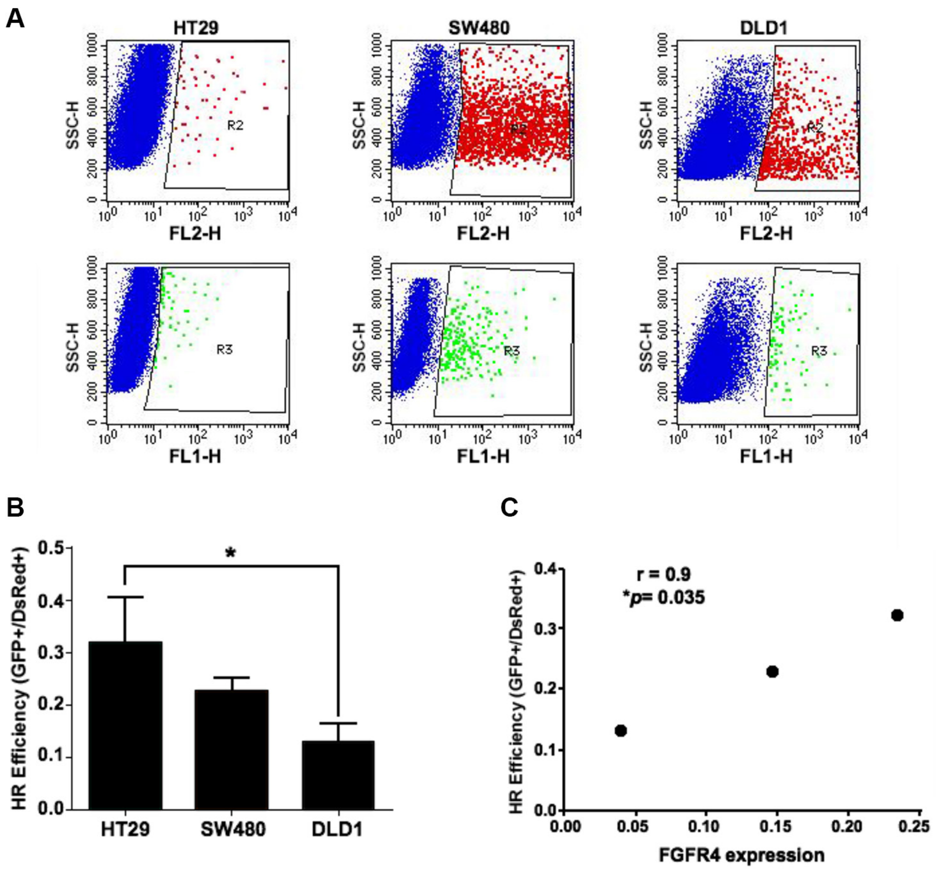

Supplementary Figure S2: (**A**) FACS analysis was performed from HT29, SW480 and DLD1 cells, using the BD FACSCalibur™ flow cytometer. Measurement of the fluorescence signals was performed 48 h after transfection of the cells with the HR construct mixture (GFP-based reporter cassette, I-SceI plasmid and DsRed-plasmid). (**B**) Quantification of the HR capacity calculated from the ratio of FL1 (GFP) and FL2 (DsRed) fluorescence intensities. The bars represent the mean of 3 independent cultures ± SEM. (**C**) Correlation of FGFR4 expression and HR capacity was calculated using GraphPad Prism software.

Details of antibodies for western blot and staining protocols

Supplementary Table S1: Primary and secondary antibodies used in the study

Name	Species	Company	Catalog#	Dilution*
Alexa Fluor® 488 secondry Ab	Rabbit	Cell Signaling	4412	1:750 (IF)
Anti-beta Actin	Mouse	Sigma	A-5441	1:3 000 (WB)
Anti-cyclin B1	Rabbit	Cell Signaling	4138	1:1000 (WB)
Anti-FGFR4 (C-16)	Rabbit	Santa Cruz	sc-124	1:100 (IHC)
Anti-FGFR4 (D3B12)	Rabbit	Cell Signaling	8562	1:1 000 (WB)
Anti-mouse TRITC secondry Ab	Mouse	Sigma	T 5393	1:250 (IF)
Anti-pFGFR (Y653/654)	Rabbit	Cell Signaling	3471	1:1 000 (WB)
Anti-phospho-cdc2 (Tyr15)	Rabbit	Cell Signaling	9111	1:1 000 (WB)
Anti-phospho-Histone H2AX (Ser139)	Rabbit	Cell Signaling	9718	1:1 000 (WB)<br>1:200 (IF)
Anti-RAD51	Mouse	Abnova	H00005888-B01P	1:250 (IF)<br>3 μg/ml (IHC)
Anti-RAD51 (D4B10)	Rabbit	Cell Signaling	8875	1:1 000 (WB)
Anti phospho-Histone H3 (Ser10)	Rabbit	Cell Signaling	9701	1:1000 (WB)
HRP-conjugated anti-mouse IgG secondry Ab	Mouse	Pierce	1858413	1:10 000 (WB)
HRP-conjugated anti-rabbit IgG secondry Ab	Rabbit	Bethyl Laboratories	A120-201P	1:20 000 (WB)

*Abbreviations are: IF = immunofluorescence, IHC = immunohistochemistry, WB = western blot.
